# A Polymeric Nanomedicine Diminishes Inflammatory Events in Renal Tubular Cells

**DOI:** 10.1371/journal.pone.0051992

**Published:** 2013-01-02

**Authors:** Álvaro C. Ucero, Sergio Berzal, Carlos Ocaña-Salceda, Mónica Sancho, Mar Orzáez, Angel Messeguer, Marta Ruiz-Ortega, Jesús Egido, María J. Vicent, Alberto Ortiz, Adrián M. Ramos

**Affiliations:** 1 Laboratory of Nephrology and Vascular Pathology, Instituto de Investigación Sanitaria-Fundación Jiménez Díaz (IIS-FJD), Madrid, Spain; 2 Department of Medicinal Chemistry, Centro de Investigación Príncipe Felipe, Valencia, Spain; 3 Department of Chemical and Biomolecular Nanotechnology, Institut de Química Avançada de Catalunya, Barcelona, Spain; 4 Cellular Biology in Renal Diseases Laboratory, Universidad Autónoma de Madrid, Madrid, Spain; 5 Fundación Renal Íñigo Álvarez de Toledo (FRIAT), Madrid, Spain; Friedrich-Alexander University Erlangen, Germany

## Abstract

The polyglutamic acid/peptoid 1 (QM56) nanoconjugate inhibits apoptosis by interfering with Apaf-1 binding to procaspase-9. We now describe anti-inflammatory properties of QM56 in mouse kidney and renal cell models.

In cultured murine tubular cells, QM56 inhibited the inflammatory response to Tweak, a non-apoptotic stimulus. Tweak induced MCP-1 and Rantes synthesis through JAK2 kinase and NF-κB activation. Similar to JAK2 kinase inhibitors, QM56 inhibited Tweak-induced NF-κB transcriptional activity and chemokine expression, despite failing to inhibit NF-κB-p65 nuclear translocation and NF-κB DNA binding. QM56 prevented JAK2 activation and NF-κB-p65(Ser536) phosphorylation. The anti-inflammatory effect and JAK2 inhibition by QM56 were observed in Apaf-1^−/−^ cells. In murine acute kidney injury, QM56 decreased tubular cell apoptosis and kidney inflammation as measured by down-modulations of MCP-1 and Rantes mRNA expression, immune cell infiltration and activation of the JAK2-dependent inflammatory pathway.

In conclusion, QM56 has an anti-inflammatory activity which is independent from its role as inhibitor of Apaf-1 and apoptosis and may have potential therapeutic relevance.

## Introduction

Acute kidney injury (AKI) and progressive loss of renal function in chronic kidney disease (CKD) are associated with interstitial inflammation and loss of tubular cells. Infiltration by leucocytes depends on the local expression of inflammatory cytokines released by renal cells. Renal tubular cells compose most of the mass of the functioning kidney and they are thought to be a central cell type in renal inflammation [1]. Tubular cells release an array of cytokines and chemokines in response to various immune and non-immune factors, contributing to attraction of inflammatory cells to the kidney [2]. Chemokines and their receptors contribute to tissue injury in animal models of inflammatory kidney disease and their therapeutic targeting decreases inflammation and tissue injury during renal disease [3]. NF-κB is a key promoter of inflammation in the kidney that integrates intracellular signals derived from many inflammatory stimuli and drives the expression of cytokines and chemokines. Pharmacological targeting of NF-κB has also been proposed as a therapy to reduce kidney damage [4]. Similar to nephrotoxins such as cyclosporine A (CsA), inflammatory cytokines may induce tubular cell injury and death [5], [6]. Cytokines belonging to the TNF superfamily may also induce an inflammatory response [7]. Thus, tissue injury leads to inflammation and inflammation may promote tissue injury. In this regard, drugs that prevent tissue injury, by protecting from apoptotic cell death, and, additionally, limit the inflammatory response, may be especially useful in kidney disease.

Apoptosis is an active process of cell death that regulates cell number [8]. Apoptosis has potential therapeutic relevance, since it is regulated by the activation of intracellular lethal molecules in response to the cell environment. Caspases are intracellular cysteine proteases that behave as activators and effectors of apoptosis, and play a central role in the process [8]. Caspases may be activated through a death receptor-dependent and a mitochondrial pathway. Death receptor-dependent apoptosis is often amplified by the mitochondrial pathway. Activation of such pathway leads to the release of proapoptotic molecules, such as cytochrome c, from mitochondria into the cytoplasm. In the presence of dATP, cytochrome c induces the formation of the Apaf-1 (apoptotic protease activating factor 1)-containing macromolecular complex called the apoptosome. This complex binds to and activates caspase-9. Mature caspase-9 activates effector caspases, leading to apoptotic cell death [9].

Recent reports have proposed the apoptosome as an interesting target for the development of apoptosis inhibitors [10], [11]. Polyglutamic acid-based nanoconjugates (PGA-peptoids) are well suited for *in vivo* applications [11]. The polymeric nanoconjugate PGA-peptoid QM56 is a first generation Apaf-1 inhibitor that prevents apoptosome formation and hence caspase activation and apoptotic cell death. Binding of procaspase-9 to Apaf-1 is mediated by caspase recruitment domain (CARD)/CARD interactions [12]. The compound action may be mediated by direct interaction with the CARD motif of Apaf-1 precluding the recruitment of procaspase-9 by Apaf-1 [11]. It has been previously demonstrated that QM56 prevents apoptosis of tumor cells induced by antitumor drugs and also protects cultured cardiomyocytes from hypoxia-induced death [13]. Moreover, QM56 protects mesothelial cells from cytokine- and toxin-induced injury (cell death and remesothelization prevention) in culture and *in vivo* [14]. In this work, we were addressed potential beneficial effects of QM56 on the kidney. While researching the possible implications of inhibition of renal cell apoptosis by QM56 we observed a potent anti-inflammatory effect. Here we describe this new anti-inflammatory effect of QM56 independent of its anti-apoptotic action that may contribute to the protection from experimental AKI afforded by the compound *in vivo*.

## Materials and Methods

### Ethics Statements

Studies were conducted in accord with the NIH Guide for the Care and Use of Laboratory Animals (NIH Publication N° 85-23, revised 1996) and was approved by the institution's animal subject review committee. Emphasis was made in the humane care of the animals.

### Cells and reagents

MCT cells are a cultured line of murine proximal tubular epithelial cells originally obtained from Eric Neilson (Vanderbildt University, Nashville, TN) that have been extensively characterized [15]. Homozygous embryonic fibroblasts from Apaf-1^−/−^ mice (MEF-Apaf-1^−/−^) and wild-type controls (MEF-Apaf-1^+/+^) [16] were generously donated by Prof Francesco Cecconi (Biology Department, University of Rome “Tor Vergata”, Rome, Italy). Both cell lines were grown as described in Materials and Methods S1. Unless otherwise specified, the concentration of Tweak (Millipore, Temecula, CA) was 100 ng/ml, murine TNFα (Peprotech, London, UK) 30 ng/ml and human interferon-γ (INFγ) (Peprotech) 30 U/ml. CsA (10 mg/ml stock solution in ethanol) (Calbiochem, La Jolla, CA) was added at a final concentration of 10 µg/ml. Based on previous experience and set-up experiments, cell death was assessed at 24 h (Tweak/TNFα/IFNγ) or 48 h (CsA) [5], [6]. The QM56 initial dose range was chosen based on published dose-response curves and dose-response studies [13], [14]. Additional dose-response studies were performed in MCT cells exposed to CsA and the concentration chosen for the studies was 20 µmol/L drug-equivalents. The JAK2 inhibitors AG490 and JAK2 Inhibitor II (50 µmol/L, Calbiochem) were used according at concentrations previously observed to offer complete protection against apoptotic challenge [17]. Additionally Western blot experiments confirmed inhibition of JAK2 phosphorylation. Parthenolide (Sigma-Aldrich, St Louis, MO) 10 µmol/L inhibits NF-κB in MCT cells without decreasing cell viability [2]. Lipopolysaccharide from E.Coli was purchased from Sigma-Aldrich.

### Synthesis of PGA-peptoid 1 conjugate QM56

The antiapoptotic polymeric nanomedicine, PGA-peptoid QM56, is the result of the conjugation of peptoid 1 to poly-L-glutamic acid (PGA) according to a standardized procedure [10] (See Materials and Methods S1). Full structural and biophysical characterization of this nanoconjugate was previously performed [10].

### Apoptosis

In renal tubular cells, apoptosis was characterized by functional and morphologic criteria [6] (See Materials and Methods S1). Cells were permeabilized and propidium iodide stained and the cell cycle analyzed by flow cytometry. Alternatively, apoptotic nuclei were observed using DAPI (Vector Laboratories, Inc., Burlingame, CA) staining. In renal tissue, apoptotic cell death was assessed by enzymatic in situ labeling of DNA strand breaks using terminal deoxynucleotidyl-transferase-mediated dUTP nick-end labeling (TUNEL) (In Situ Cell Death Detection Kit; Promega, Madison, WI).

### Quantitative reverse transcription-polymerase chain reaction

One µg RNA isolated by Trizol (Life Technologies, Carlsbad, CA) was reverse transcribed with High Capacity cDNA Archive Kit and real-time PCR was performed on a ABI Prism 7500 PCR system (Applied Biosystems, Foster City, CA) using the DeltaDelta Ct method. Expression levels are given as ratios to GAPDH. Pre-developed primer and probe assays were from Applied Biosystems.

### ELISA

Cells were stimulated with 100 ng/mL Tweak and murine MCP-1 in the supernatants was determined by ELISA (BD Pharmingen, NJ) according to manufacturer's instructions.

### Immunostaining

Cells plated onto Labtek™ slides were fixed in 4% paraformaldehyde and permeabilized in 0.2% Triton X-100/PBS, washed in PBS and incubated with rabbit polyclonal anti-RelA (1∶75) (Santa Cruz Biotechnology, Santa Cruz, CA), rabbit policlonal anti-phospho STAT3-(Ser727)(1∶100) (Cell Signaling Technology, Danvers, MA) or rabbit monoclonal antibody anti-phospho-NF-κB p65 (Ser536)(1∶100) (Cell Signaling Technology, Danvers, MA) followed by FITC secondary antibody (1∶200, Sigma-Aldrich, Spain). Nuclei were counterstained with propidium iodide.

### Electrophoretic mobility shift assay (EMSA)

Cells were resuspended and homogenized in extraction buffer (see Materials and Methods S1) and EMSA was carried out as previously described [2].

### NF-κB Luciferase Reporter Assay

MCT cells were plated at a density of 8×10^4^ cells in six-well plates 24 h before transfection with FuGENE 6 (Roche, Indianapolis, IN), according to the manufacturer's intructions. pNF-κB-Luc (Stratagene, La Jolla, CA) and pRLTK vectors, which contains the luciferase gene Renilla (Promega), were used in a ratio of 10∶1. The medium was replaced with RPMI without serum 4 h after transfection and cells were treated with the stimuli. Luciferase activity was determined by a luciferase assay system (Promega) and a luminometer (Berthold, Nashua, NH) and normalized to Renilla activity to control for differences in transfection efficiency.

### Western blot

Standard procedures were applied (See Materials and Methods S1) [5]. The following primary antibodies were used: anti p65 and anti-JAK2 (Santa Cruz Biotechnology), anti phospho JAK2 (pYpY1007 1008) (Invitrogen, Camarillo, CA) and anti-phospho Stat 3 (Ser727) and anti-Stat 3 (Cell Signaling Technology) rabbit polyclonal antibodies; anti-phospho-NF-κB p65 (Ser536) and anti-phospho IκBα (Cell Signaling Technology) rabbit monoclonal antibody; anti-Apaf-1 (BD Pharmingen) and anti-α-Tubulin (Sigma-Aldrich) mouse monoclonal antibody; anti-GAPDH (Chemicon International, Temecula, CA) mouse polyclonal antibody.

### Animal model

Folic acid nephropathy is a classical model of AKI [18]–[20]; C57/BL6 mice from 12- to 14-week-old (IFFA-CREDO, Barcelona, Spain) received a single intra-peritoneal injection of folic acid (Sigma-Aldrich) 250 mg/kg in 0.3 M sodium bicarbonate or vehicle and mice were killed 24 h later. A single dose of QM56 (100 mg/Kg) was intravenously administered 6 hour before folic acid. Groups (n = 8–10) were as follows: a) Healthy control (folic acid vehicle), b) AKI (Folic acid plus QM56 vehicle), c) AKI plus QM56. Blood was drawn for urea and creatinine assessment and the kidneys were perfused in situ with cold saline before removal. One kidney was snap-frozen in liquid nitrogen for protein and RNA studies and the other fixed and paraffin embedded.

### Immunohistochemistry

Paraffin-embedded sections were stained using standard histology procedures. Immunostaining was carried out in 3 µm thick tissue sections that were deparafinized and antigen retrieved using the PT Link system (Dako Diagnostics, Barcelona, Spain) with Sodium Citrate Buffer (10 mM) adjusted to pH 6 or pH 9 depending on the immunohystochemical marker. Inmmunohistochemical staining was performed using the Dako Autostainer (Dako Diagnostics). Briefly: endogenous peroxidase was blocked and then sections were incubated for 20–30 min at room temperature with primary antibody: rabbit polyclonal anti active caspase 3 (Promega), and monoclonal mouse anti CD68 and rabbit polyclonal anti-CD3 (Dako Diagnostics) ready to use. After washing, slides were treated with the EnVision™ DuoFLEX Doublestain System using 3,3′-diaminobenzidine as cromogen. Sections were counterstained with Carazzi's hematoxylin. The total number of caspase 3 stained tubuli was counted in 10 randomly chosen fields (200×) and the result expressed as the Mean±SD of positive tubuli per field. The total number of CD68 and CD3 positive stained cells was quantified in at least 5 randomly chosen fields (200×) using Image-Pro Plus software. Data are expressed as positive stained area vs. total analyzed area. Samples from each animal were examined in a blind manner.

### Statistics

Statistical analysis was performed using SPSS 11.0 (SPSS, Chicago, IL). Results are expressed as mean ± SD. Significance at the p<0.05 level was assessed by non-parametric Mann-Whitney test for two groups of data and Kruskal-Wallis for three of more groups.

## Results

### QM56 inhibits apoptosis and expression of chemokines induced by different cell death stimuli in renal tubular epithelial cells

**Figure 1:**
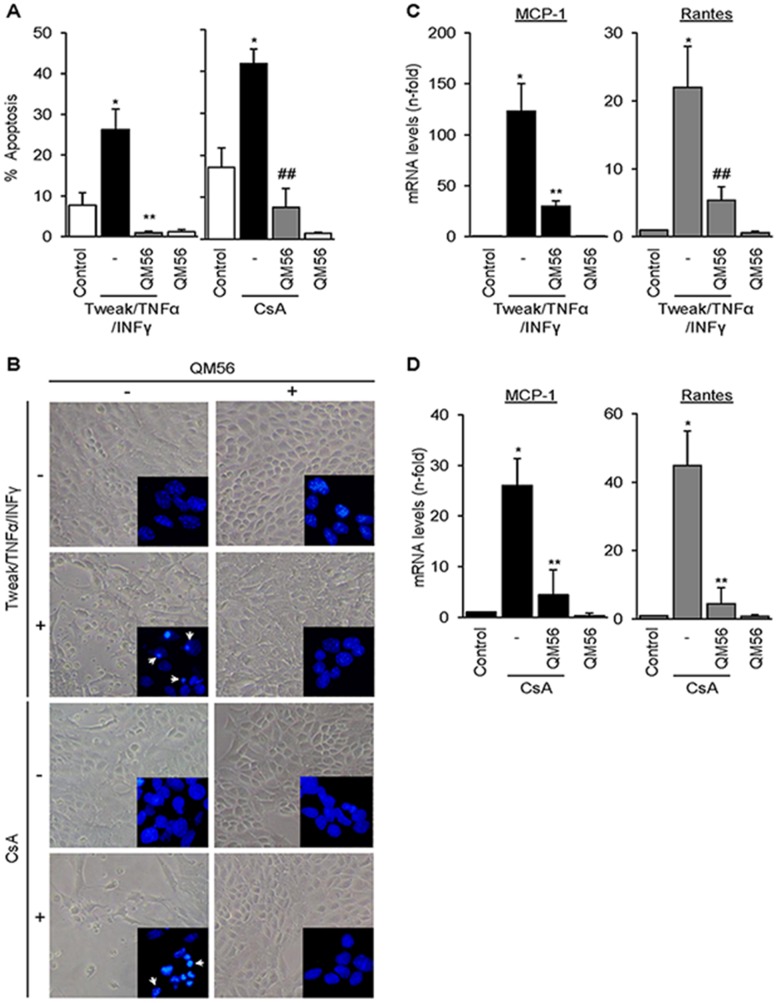
QM56 protects tubular cells from apoptosis and prevents the expression of CsA and cytokines-induced chemokines. **A**) MCT tubular cells were cultured in the presence of 100 ng/ml Tweak, 30 ng/ml TNFα, 30 U/ml IFNγ for 24 h or in the presence of 10 µg/ml CsA for 48 h. QM56 (10 µM) was added 1 h before lethal stimuli. Cell death was assessed by the DNA content of cell population stained with propidium iodide followed by flow cytometry. The percentage of cells with sub-G1 DNA content (hypodiploid cells) is considered as apoptotic (*p<0.01 vs Control; **p<0.01 vs Tweak/TNFα/INFγ; ##p<0.05 vs CsA). **B**) Contrast phase microscopy and assessment of nuclear morphology in MCT cells stained with DAPI (inset images) confirmed protection from apoptosis by QM56. Arrows indicate typical apoptotic cells with characteristic shrunk, pyknotic and fragmented nuclei present among Tweak/TNFα/INFγ- and CsA-treated cells but not among control cells. **C**) QM56 prevents the upregulation of MCP-1 and Rantes mRNA (qRT-PCR) in MCT cells exposed to Tweak/TNFα/INFγ for 3 h. (*p<0.01 vs Control, **p<0.05 and ##p<0.01 vs Tweak/TNFα/INFγ). **D**) QM56 prevents the upregulation of MCP-1 and Rantes mRNA in MCT cells exposed to CsA for 6 h (*p<0.05 vs Control, **p<0.05 vs CsA). Results are expressed as the Mean±SD of at least five independent experiments.

Tubular cell apoptosis and inflammation contribute to the pathogenesis of AKI and CKD. Lethal cytokines, in particular the association Tweak/TNFα/IFNγ, and the exogenous nephrotoxin CsA are well-characterized tubular cell death inducers. Tweak/TNFα/IFNγ activates the intrinsic apoptotic pathway following caspase 8 activation, while CsA directly targets the mitochondria [5], [6]. As expected from its known action in other cell systems, QM56 protected cultured renal tubular cells from apoptosis induced by the lethal cytokine cocktail Tweak/TNFα/IFNγ or by CsA (Figure 1, A and B). Interestingly, both apoptotic stimuli also induced the rapid expression of MCP-1 and Rantes mRNA and this was prevented by QM56 (Figure 1, C and D). The effect of QM56 on chemokine expression was rather unexpected and encouraged us to investigate the possible anti-inflammatory properties of QM56 and their relationship with protection from apoptosis. Prevention of chemokine expression preceded in time the protection from apoptosis, suggesting that both actions of QM56 might be independent from each other. Thus, for further experiments we chose Tweak as a stimulus, since in this cell system Tweak promotes inflammation, but not apoptosis, and thus we could dissociate effects related to apoptosis from those related to inflammation.

### QM56 inhibits expression of chemokines induced by Tweak, a non-lethal stimulus

**Figure 2:**
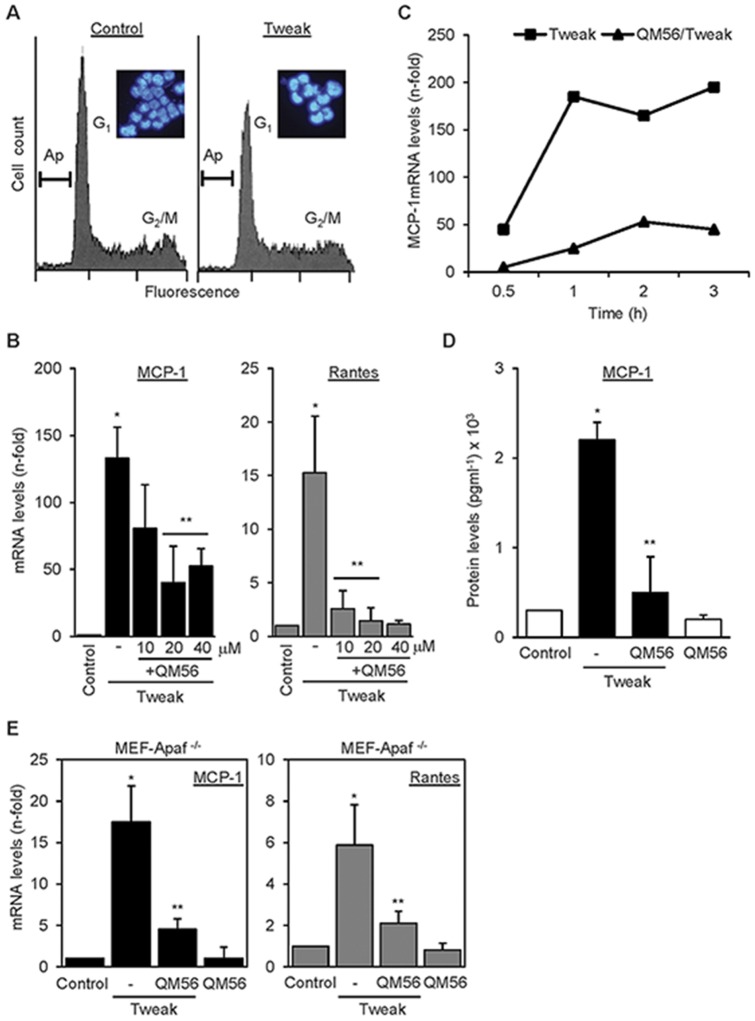
QM56 prevents the expression of inflammatory mediators induced by the non-lethal stimulus Tweak. **A**) Treatment with 100 ng/ml Tweak for 24 h does not promote apoptosis (Ap) in MCT cultured tubular cells. Apoptotic cells were measured by flow cytometry analysis of DNA content in cells stained with propidium iodide and the cell nuclear morphology through DAPI staining followed by phase contrast microscopy as in Fig. 1 (inseted images). **B**) QM56 dose-dependently prevents the upregulation of MCP-1 and Rantes. qRT-PCR in MCT cells exposed to Tweak for 3 h. Mean±SD of three independent experiments. *p<0.05 vs Control, **p<0.05 vs Tweak. **C**) QM56 prevents the upregulation of MCP-1 in MCT tubular cells in response to 100 ng/ml Tweak from very early time points. Representative experiment. **D**) QM56 prevents the upregulation of MCP-1 (ELISA) in supernatants from MCT cells treated with 100 ng/ml Tweak. Mean±SD of three independent experiments.*p<0.05 vs Control, **p<0.05 vs Tweak. **E**) QM56 prevents the upregulation of MCP-1 and Rantes mRNA in MEF-Apaf-1^−/−^ cells stimulated with Tweak. (*p<0.01 vs control,**p<0.01 vs Tweak). Mean±SD of four independent experiments.

Tweak is a mediator of tubulointerstitial inflammation in AKI that does not promote apoptosis in non-stressed renal tubular cells [2], [6]. We confirmed the lack of lethality of Tweak in the present experiments (Figure 2.A). In this system, in the absence of cell death, QM56 prevented Tweak-induced upregulation of MCP-1 and Rantes mRNA expression in a dose-dependent manner (Figure 2B). In most experiments cells were stimulated by Tweak for 3 hours. However, time-course experiments demonstrated that QM56 prevented an increase in chemokine mRNA expression from as early as 30 minutes following Tweak stimulation (Figure 2C). QM56 also reduced chemokine secretion to the culture supernatant in response to Tweak, as exemplified by MCP-1 (Figure 2D). These results indicate that the anti-inflammatory effect of QM56 is observed in an experimental system where inflammation was triggered by a stimulus that did not promote apoptosis.

### QM56 inhibits inflammation in Apaf-1 null cells

In order to test whether the anti-inflammatory action of QM56 was dependent on its ability to inhibit Apaf-1, we tested Apaf-1 knock-out murine embryonic fibroblasts (MEF-Apaf-1^−/−^). It has been previously described that these cells are protected from pro-apoptotic stimuli [16]. Gene and protein expression analysis confirmed lack of Apaf-1 in these cells (Figure S1, A and B). Tweak promoted the expression of MCP-1 and Rantes mRNA in MEF-Apaf-1^−/−^ and this effect was prevented by QM56 (Figure 2E). This result suggests that inhibition of the inflammatory response by QM56 does not involve Apaf-1.

### QM56 does not prevent early NF-κB activation induced by Tweak in tubular cells

**Figure 3:**
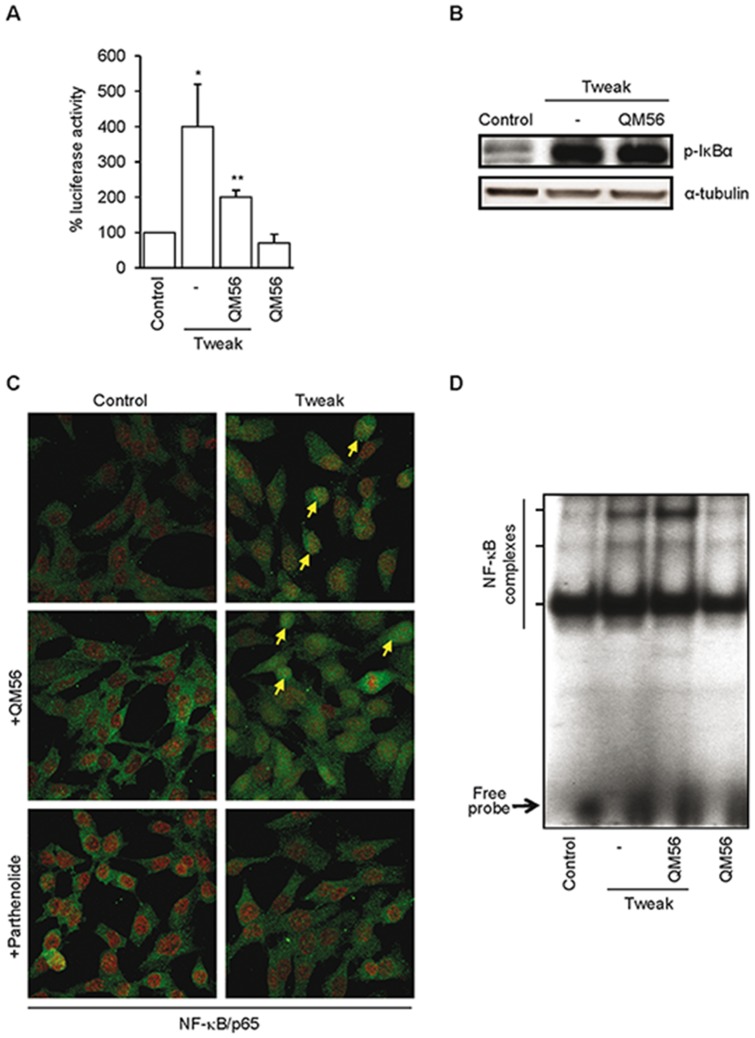
QM56 prevents Tweak-induced NF-κB-dependent transcription, but not NF-κB p65/relA nuclear translocation or NF-κB DNA-binding. **A**) Tweak-induced NF-κB transcriptional activity in MCT cells is inhibited by QM56. NF-κB transcriptional activity was expressed as a percentage of Luciferase activity normalized to Renilla activity in a reporter gene assay. *p<0.01 vs Control; **p<0.05 vs Tweak (n = 3). **B**) Tweak increases IκBα phosphorylation after 30 minutes of incubation and pretreatment with QM56 does not preclude this effect. Representative Western blots are shown (n = 3). Protein bands in the sequence were arranged from non-consecutive lanes on the same membrane. **C**) QM56 does not prevent NF-κB p65/relA nuclear translocation in response to Tweak. MCT cells were stimulated for 30 minutes with the stimuli alone or in the presence of QM56 added 1 h before stimulation. Confocal microscopy: p65 (green), propidium iodide (red). Parthenolide was used as a control inhibitor of p65 nuclear translocation. Arrows indicate individual cells shown nuclear localization of p65 NF-κB subunit. **D**) QM56 does not prevent NF-κB DNA-binding activity in response to Tweak. Representative EMSA of nuclear extracts from MCT cells treated with Tweak alone or pretreated with QM56.

The pro-inflammatory effect of Tweak on MCP-1 and Rantes expression is mediated by NF-κB activation and p65/relA translocation to the nucleus [2]. In this regard, the inhibitor of Iκ-Bα degradation parthenolide [21], prevented Tweak-induced NF-κB p65/RelA nuclear translocation, NF-κB DNA-binding activity, NF-κB transcriptional activity and upregulation of MCP-1 and Rantes mRNA in tubular cells [2]. We tested whether QM56 may inhibit some of these steps. QM56 prevented Tweak-induced upregulation of NF-κB-dependent genes from very early time points (Figure 2C) and repressed the overall NF-κB transcriptional activity measured by gene reporter assay (Figure 3A). However, neither IκBα phosphorylation events nor the consequent NF-κB p65/relA nuclear translocation in response to Tweak in tubular cells were observed to be inhibited (Figure 3B and 3C). Furthermore, QM56 did not prevent nuclear NF-κB DNA-binding in response to Tweak, as assessed by EMSA (Figure 3**D**).

### Inhibition of JAK2 prevents Tweak-induced chemokine mRNA expression in tubular cells and MEFs

**Figure 4:**
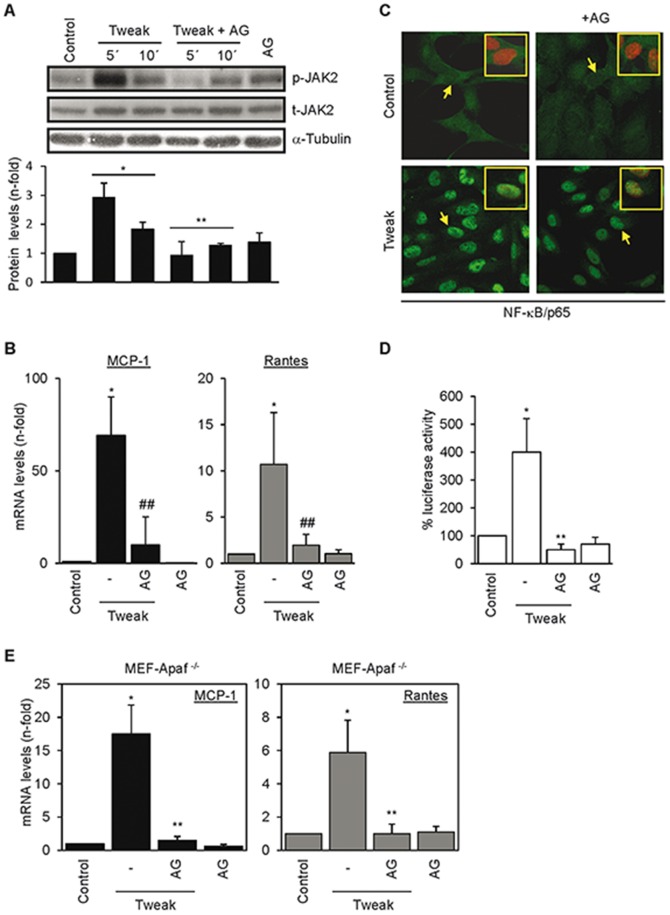
Regulation of Tweak induction of chemokine mRNA by chemical inhibition of JAK2 kinase. **A**) Tweak promotes JAK2 phosphorylation in MCT cells which is prevented by the JAK2 inhibitor AG490 (AG). *p<0.05 vs Control; **p<0.05 vs Tweak (n = 3). **B**) Influence of JAK2 (AG) inhibitor on Tweak-induced expression of MCP-1 and Rantes (*p<0.01 vs Control; ^##^p<0.01 vs Tweak) mRNA in MCT cells. Mean±SD of at least three independent experiments. **C**) Inhibition of JAK2 with AG490 does not prevent Tweak-induced p65/RelA nuclear translocation. MCT cells were stimulated for 30 minutes with the stimuli alone or in the presence of AG490 (AG) added 1 h before stimulation and then analyzed by confocal microscopy. Arrows point to cells shown amplified as merge images in inset (p65 (green), propidium iodide (red)). **D**) Tweak-induced NF-κB transcriptional activity is inhibited by JAK2 inhibition in MCT cells. NF-κB transcriptional activity was expressed as a percentage of Luciferase activity normalized to Renilla activity in a reporter gene assay. *p<0.01 vs Control; **p<0.01 vs Tweak (n = 3). **E**) AG490 (AG) prevents the Tweak-stimulated expression of MCP-1 and Rantes mRNA in MEF-Apaf-1^−/−^ cells. *p<0.01 vs Control, **p<0.01 vs Tweak. Mean±SD of three independent experiments.

Protein phosphorylation regulates multiple steps in signal transduction and gene transcription. The JAK/STAT pathway mediates renal damage and inflammation [17], [22]. While studying the role of JAK/STAT in kidney injury we observed that Tweak induced phosphorylation of JAK2 and its downstream substrate Stat-3 which were inhibited by AG490 in tubular cells (Figure 4A) and chemical inhibition of JAK2 by AG490 prevented Tweak-induced chemokine mRNA expression (Figure 4B). The JAK2 inhibitor II also prevented Tweak-induced upregulation of MCP-1 and Rantes mRNA levels (25% MCP-1 and Rantes mRNA expression compared to 100% MCP-1 and Rantes mRNA expression in presence of Tweak alone; p<0.01, not shown). Similar to QM56, AG490 did not interfere with Tweak-induced NF-κB nuclear translocation (Figure 4C), but repressed the overall NF-κB transcriptional activity measured by gene reporter assay (Figure 4D). Furthermore, like QM56, AG490 prevented Tweak-induced chemokine mRNA expression in MEF-Apaf-1^−/−^ (Figure 4E). The similarity between the effects of AG490 and QM56 suggested that QM56 may inhibit JAK2 activity and we tested this hypothesis.

### QM56 inhibits JAK2 in tubular cells and MEFs

**Figure 5:**
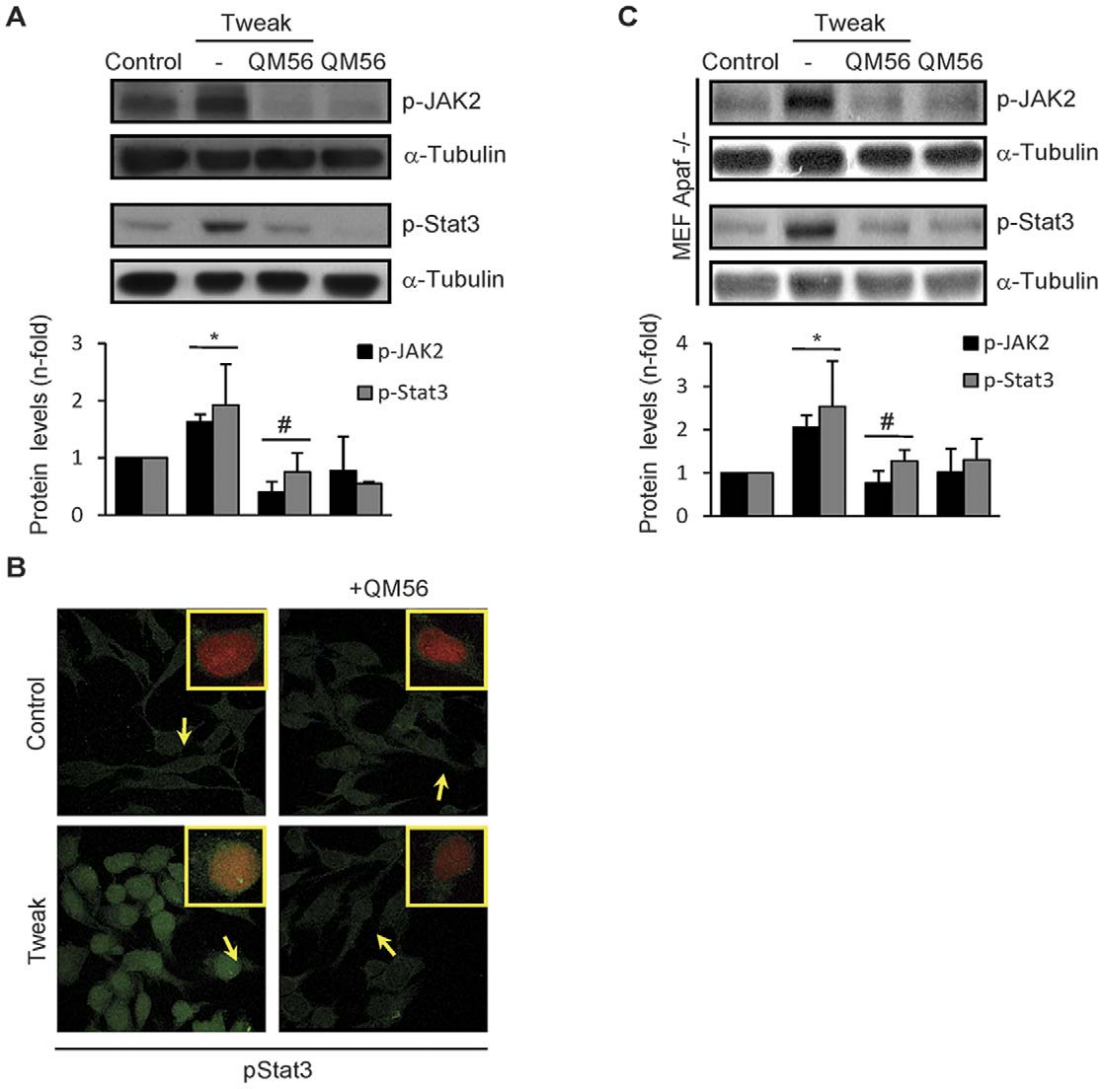
QM56 inhibits Tweak-dependent JAK2/STAT-3 pathway activation. MCT (A and B) and MEF-Apaf-1^−/−^ (C) cells were treated with 100 ng/ml Tweak, alone or pretreated with QM56 (20 µmol/L) for 1 h before Tweak addition. JAK2 and STAT-3 phosphorylation were evaluated by Western blot in cells stimulated with Tweak for the indicated times. Phosphorylated STAT-3 was also evaluated by confocal microscopy 40 min. following Tweak stimulation. The figures are representative of three independent experiments. **A**) QM56 prevents Tweak-induced JAK2 activation and the downstream STAT-3 phosphorylation. Bands in the sequence were arranged from non-consecutive lanes on the same membrane. *p<0.05 vs Control; ^#^p<0.05 vs Tweak (n = 3). **B**) QM56 also prevents the STAT-3 (green) nuclear translocation elicited by Tweak. Arrows indicate representative nuclei of each culture condition that are shown in detail in a merged green-propidium iodide image inset in each treatment photograph. **C**) QM56 hinders Tweak-induced JAK2/STAT3 phosphorylation in cells lacking Apaf-1 (MEF-Apaf-1^−/−^).*p<0.05 vs Control; ^#^p<0.05 vs Tweak (n = 3).

QM56 prevented Tweak-induced phosphorylation of JAK2 and its downstream target STAT3 (Figure 5A) and pSTAT3 nuclear translocation in tubular cells (Figure 5B). Tweak stimulation also resulted in JAK2 and STAT3 phosphorylation in MEF-Apaf-1^−/−^ which was prevented by QM56 (Figure 5C). The inhibitory action of QM56 on JAK2 was also observed in cells treated with CsA and Tweak/TNFα/INFγ (Figure S2). These results suggest that inhibition of JAK2 activation could contribute to the observed anti-inflammatory effect of QM56.

### QM56 interferes with activating posttranslational modification of NF-κB

**Figure 6:**
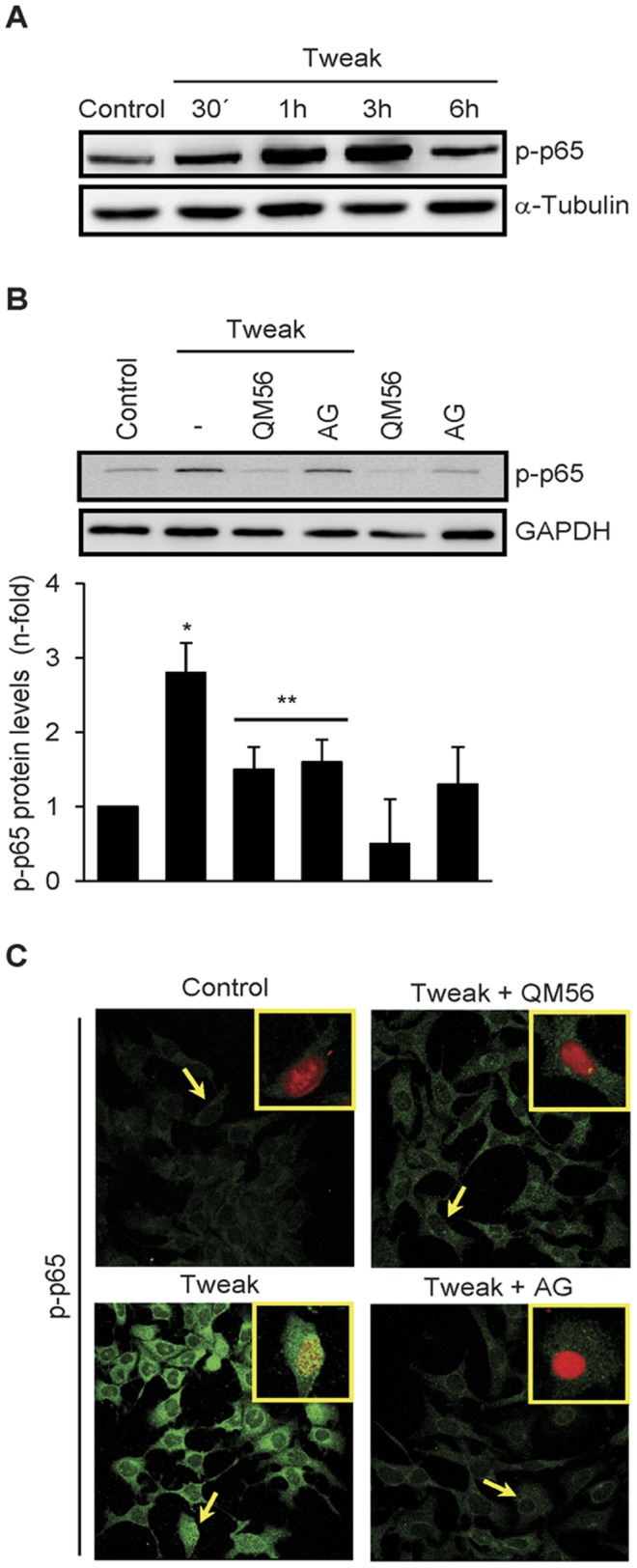
QM56 and JAK2 inhibition negatively regulate Tweak-induced p65 phosphorylation. **A**) Tweak phosphorylates p65. MCT cells were treated with 100 ng/ml Tweak for the indicated times and phosphorylation of p65 in the Ser536 analyzed by Western blot. Representative figure of three individual experiments. **B**) QM56 and JAK2 inhibition prevent p65 phosphorylation. MCT cells were treated with 100 ng/ml Tweak for 3 h in the absence or in the presence of QM56 or AG490 (AG) added 1 h before the cytokine. Phosphorylated p65 was analyzed by Western blot. The figure shows a representative experiment and quantitation as Mean±SD of five independent experiments (*p<0.01 vs Control, **p<0.05 vs Tweak). **C**) QM56 and AG490 (AG) inhibit p65(Ser536) phosphorylation and nuclear translocation. MCT cells were treated as in B. Phospho-p65(Ser536) was studied by confocal microscopy. Arrows indicate representative cells with nuclear location of phospho-p65(Ser536) which are shown in detail in a merged green-propidium iodide image inset.

Phosphorylation of transcription factors modulates their transcriptional activity [23]. Phosphorylation of Ser536 on p65 has been related to transcriptional control of NF-κB-regulated inflammatory genes [24], [25]. Tweak time-dependently increased p65 phosphorylation at Ser536 (Figure 6A). This was significantly attenuated by QM56 or AG490 (Figure 6B). Moreover, Tweak-induced phospho-p65 (Ser536) nuclear translocation was inhibited by QM56 or AG490 (Figure 6C). Finally, to address whether NF-κB-dependent chemokines synthesis inhibition by QM56 was stimulus dependent, we treated tubular cells with LPS as another representative NF-kB agonistic activity. Whereas LPS activated JAK2/STAT3/p-p65 pathway and consequently induced chemokine expression, QM56 significantly prevented or reduced LPS-engaged signaling and resulting inflammatory events (Figure S3). These results demonstrate that JAK2 modulates p65 Ser536 phosphorylation and suggest that QM56 may interfere with the complete activation of NF-κB-dependent chemokine gene expression through inhibition of JAK2 and p65 (Ser536) phosphorylation.

### QM56 reduces renal chemokine gene expression during AKI

**Figure 7:**
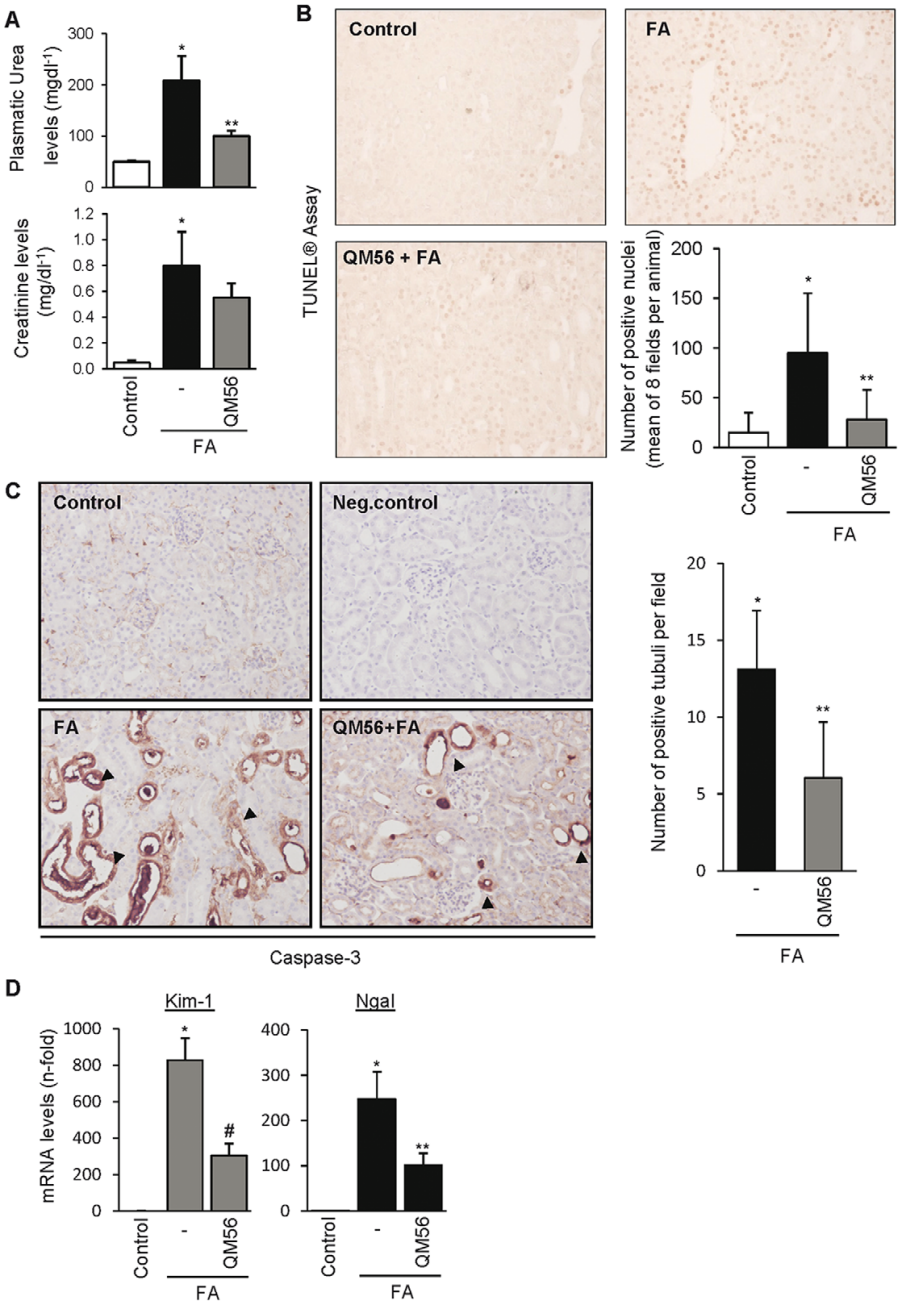
QM56 reduces the degree of renal disfunction and damage in acute kidney injury. AKI was induced by a single folic acid (FA) overdose and mice were killed at 24 h. QM56 decreased the magnitude of renal failure as assessed by: plasma urea and creatinine (*p<0.01 vs Control, **p<0.05 vs FA) (**A**), tubular cell apoptosis as assessed by TUNEL® (*p<0.01 vs Control, **p<0.05 vs FA) and immunohistochemistry of caspase-3 (*p<0.01 vs Control, **p<0.02 vs FA) (**B,C**) and Kim-1 and Ngal mRNA hindered synthesis (*p<0.01 vs Control, ^#^p<0.01 vs FA, **p<0.05 vs FA) (**D**). In C, black arrows indicate caspase-3 positive tubuli. Control for the technique (negative control, NC) is stained in the absence of primary antibody. Magnifications: ×200. Mean±SD of 8–10 mice per group.

**Figure 8:**
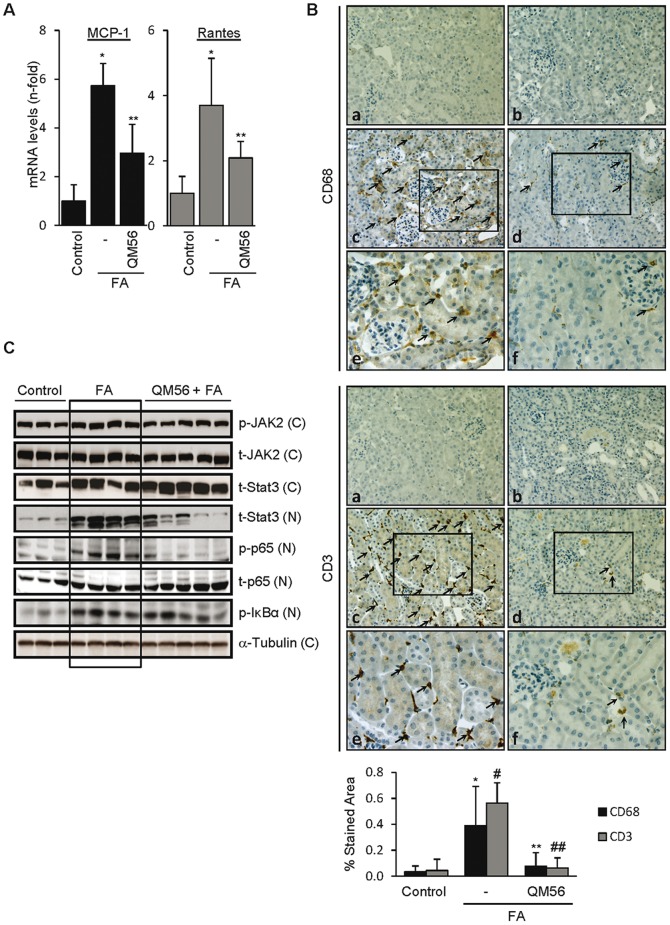
QM56 reduces renal inflammation in acute kidney injury. **AKI was induced by a single folic acid (FA) overdose and mice were killed at 24 h**. **A**) renal expression of MCP-1 and Rantes as assessed by qRT-PCR (*p<0.05 vs Control, **p<0.05 vs FA). **B**) The increased number of interstitial macrophage stained with anti CD68 (left panel) or CD3 (right panel) in AKI kidneys was reduced by QM56. Representative images of groups of mice control (a) treated with FA (c and detail in e) and treated with FA and QM56 (d and detail in f). Controls for the technique were stained in the absence of primary antibodies (b). Arrows on c, d, e and f indicate macrophage infiltration (left panel) and lymphocyte infiltration (right panel). Delimited area in c and d are correspondingly amplified in e and f, respectively. Original magnifications ×200 and details ×400. Quantification as Means±SD of 5 mice per group (*p<0.02 vs Control, **p<0.02 vs FA, ^#^p<0.01 vs Control, ^##^p<0.01 vs FA) **C**) Western blots of nuclear (N) and cytoplasmatic (C) mouse kidney extracts from representative untreated control and FA and FA+QM56-treated mice. The phosphorylated form of JAK2 is increased in the folic acid-induced AKI group (1.4±0.1 fold increase vs Control group, p<0.02) and reduced by treatment with QM56 (1.1.±0.1 fold increase vs Control group, p<0.01 vs FA group). JAK2 activation induces both STAT3 nuclear translocation and phosphorylation of p65 in nuclei and they were prevented by QM56. However, IκBα phosphorylation and p65 nuclear translocation were not affected by QM56.

In order to assess the potential implications of the observed action of QM56 in cultured tubular cells, QM56 was tested in an animal model of AKI that shares features with human AKI, such as tubular cell apoptosis and inflammation [18]–[20], [26]. AKI was induced by a single folic acid overdose. QM56 seemed to ameliorate renal function of FA-treated mice as judged by lower plasma urea and creatinine levels showed by the QM56-treated group (Figure 7A). Moreover, QM56 also decreased tubular cell DNA fragmentation and caspase-3 expression, both of them indicative of cell apoptosis (Figure 7, B and C). Remarkably, QM56-treated mice also displayed improved tubular damage condition as was evaluated by a significantly lowered mRNA synthesis of the early and sensitive AKI markers Kim-1 and Ngal (Figure 7D). Mice treated with QM56 also shown a significantly reduced renal inflammation as was evaluated by MCP-1 and Rantes mRNA expression (Figure 8A) and decreased macrophage and lymphocyte infiltration (Figure 8B). We also studied the regulation of key proinflammatory signaling events we previously showed to be targeted by QM56 in tubular cells (Figure 8C). The mice AKI group displayed increased phospho JAK2 levels compared to the control group. Accordingly, FA treated mice exhibited both higher STAT3 and phosphorylated p65 nuclear levels, accounting for previous phosphorylation-activation events and p65 translocation, respectively. Furthermore, both JAK2/STAT3 pathway activation and p65 phosphorylation were significantly decreased in the QM56-treated mice group. However, although QM56 did restrain NF-κB-dependent proinflammatory gene synthesis, it did not avoid IκBα p65-dependent p65 nuclear translocation as was the case in cultured cells. Besides, supporting this latter result, we also observed increased tubular activation of p65 in folic acid induced AKI that was not affected by QM56 treatment. Otherwise, folic acid-induced p65 phosphorylation was decreased by QM56 (Figure S4).

## Discussion

The main finding of this study is that the QM56 nanoconjugate has a previously non-described anti-inflammatory effect, independent of cell death or Apaf-1, which may be mediated, at least in part, by inhibition of JAK2 phosphorylation. In addition, we have shown for the first time that QM56 protects cultured renal tubular cells from apoptosis induced by nephrotoxins and inflammatory cytokines. Thus, QM56 is a dual inhibitor of cell death and of inflammation and these actions may underlie its protective effect against AKI *in vivo*.

AKI is characterized by tubular cell death that is amplified by an inflammatory response. Thus, an agent that directly inhibits cell death and inflammation is a welcomed addition to the preclinical drug pipeline for AKI. In this regard, in nephrotoxic AKI induced by a folic acid overdose, a model that shares the main pathogenic processes of cell death and proliferation, inflammation and fibrosis with human AKI, results suggest that QM56 may improve renal function and tissue injury evaluated by classical clinical parameters, as well as through expression of more reliable biomarkers of AKI such as Kim-1 and Ngal [2], [26], [27]. In this model, QM56 prevented the early occurrence of apoptotic cell death as well as inflammation, the latter detected through lesser both chemokine induction and immune cell infiltration in the kidney.

*In vivo* studies do not allow easy differentiation between direct inhibition of inflammation, inhibition of cell death-generated inflammation and inhibition of inflammation-generated cell death. However, cell culture studies allow exploring the anti-inflammatory mechanisms of QM56 in addition to the anti-apoptotic properties that had been previously characterized in non-renal cells. QM56 protected from apoptosis induced either by exogenous nephrotoxic agents, such as CsA, or by endogenous inflammatory mediators that contribute to inflammation-induced apoptosis in AKI, such as the cytokine combination Tweak/TNFα/IFNγ [6]. CsA is a key immunomodulatory drug whose use in clinical transplantation is limited because of nephrotoxicity [28]. QM56 prevented both apoptosis and the expression of chemokine mRNA induced by CsA or cytotoxic cytokines. The rapid inhibitory action of QM56 on the inflammatory response elicited by CsA or Tweak/TNFα/INFγ strongly suggested this effect was not attributable to distal inhibition of apoptosis through Apaf-1 binding. To unravel whether the limitation of the inflammatory response by QM56 was related to its cytoprotective effect, we focused our studies on Tweak. Tweak is a TNF superfamily cytokine that, by itself, in the absence of additional mediators of inflammation, does not promote tubular cell apoptosis [6], as confirmed in this manuscript. QM56 also inhibited the inflammatory response induced by this non-cytotoxic cytokine. Indeed, inhibition of chemokine mRNA expression was observed as early as 30 minutes following addition of Tweak, suggesting a direct anti-inflammatory action of QM56. Since NF-κB is a key transcription factor in inflammation and had been shown to mediate Tweak-induced transcription of chemokine mRNA in tubular cells [2], [4], we explored whether QM56 interfered with NF-κB activation. Both MCP-1 and Rantes are canonical p65/RelA targets [29]. QM56 prevented transcriptional activation by NF-κB but we could not demonstrate inhibition of p65/RelA translocation to the nucleus or generation of DNA-binding NF-κB complexes. Interestingly, cells treated with AG490, displayed a similar pattern of inhibition of Tweak-induced, NF-κB-dependent transcription of chemokine-encoding genes and of a reporter gene assay, without inhibition of RelA translocation to the nucleus. AG490 is a tyrphostin JAK2 inhibitor [30], so we explored in depth the influence of QM56 on JAK2 activation.

Active JAK2 promotes phosphorylation and activation of the STAT family of transcription factors [31]. The JAK2/STAT3 pathway has been linked to generation of inflammation [32]–[38]. Although Tweak activation of JAK2 had not been reported previously, another TNF superfamily receptor, TNFR1, associates with JAK2 and TNFR1/JAK2 activates downstream kinases [37]. JAK2 may promote MCP-1 transcription and JAK2 targeting impaired the induction of MCP-1 by TNFα or IL-6 [33], [35], [36], [38]. RANTES may also be regulated by JAK2 signaling [34]. Recent evidence suggests the existence of connecting points between NF-κB and JAK/STAT signaling pathways [34], [37]. Both NF-κB and JAK2/STAT3 signaling is required for induction of MCP-1 or Rantes mRNA expression induced by Cardiotrophin-1 in HUVEC or gangliosides in brain microglial cells [34], [37], [39], [40]. Here, we demonstrated for the first time that Tweak activates JAK2 and STAT3 at very early time points and that QM56 prevents this activation, consistently with the early effect of QM56 on chemokine expression.

Kinases may phosphorylate regulatory proteins upstream of NF-κB as well as NF-κB itself and modulate NF-κB function [23]. We have now identified a Tweak-dependent JAK2 activity required for NF-κB phosphorylation at Ser536 and for NF-κB-dependent MCP-1 and Rantes transcription. Phosphorylation of Ser536 is independent from IκBα activity and steers the transcription of a set of inflammatory genes without affecting nuclear translocation, DNA binding of p65 or the general activation of NF-κB [24]. Both AG490 and QM56 prevented Tweak-induced NF-κB p65 phosphorylation at Ser536. This may explain QM56 prevention of Tweak-induced upregulation of NF-κB-dependent chemokine mRNA expression in the absence of changes in nuclear translocation of NF-κB p65 and NF-κB DNA binding. Supporting the hypothesis that only certain p65-dependent genes would be regulated by p65 phosphorylation at Ser536, QM56 or AG490 failed to prevent TWEAK-induced, NF-κB p65-dependent Klotho mRNA downregulation (unpublished observation) [41]. Furthermore, we additionally proved that QM56 also prevented LPS agonistic activity on NF-κB and inflammation by inhibiting the JAK2/STAT3 pathway and p65 phosphorylation, so confirming that the anti-inflammatory effect of QM56 is stimulus-independent and restricted to the JAK2-dependent signaling pathway. Moreover, the JAK2/STAT3 and NF-κB/p-p65 proinflammatory pathways were revealed to be active in kidneys from mice treated with folic acid and further repressed by QM56.

QM56 was originally described as an Apaf-1 CARD binding chemical inhibitor [10], [11]. However QM56 inhibited JAK2 and STAT-3 phosphorylation and chemokine mRNA expression in response to Tweak in Apaf-1 null cells, suggesting additional targets for the anti-inflammatory action of QM56. QM56 is a polymeric compound that integrates the first generation of Apaf-1 inhibitors directly derived from a library screening [11]. The CARD domain is a protein-binding motif that interacts with caspases through CARD-CARD interactions. CARD domains have been found in many adaptor proteins not related to apoptosis that mediate inflammatory responses and may regulate NF-κB signaling [42]. However, the modulation of additional intracellular inflammation signaling pathways by CARD-containing proteins has been less characterized. The CARD containing NLRP1 protein has a crucial role in the activation of pro-inflammatory caspases through the formation of the inflammasome but also modulates Apaf-1 function through CARD/CARD interactions [43], [44], suggesting the presence of a crosstalk between pathways. At present it is not known whether a molecule that binds to Apaf-1 CARD could have some residual affinity to bind CARD domains of other protein families, including those relevant for inflammatory processes. Improved generations of Apaf-1 inhibitors with restricted chemical conformations that would improve specific CARD-domain selectivity are being developed. In the same manner, QM56 offers an interesting platform to further develop inflammation targeting inhibitors.

In conclusion, the QM56 nanoconjugate has a novel, apoptosis-independent, anti-inflammatory action that appears at least in part related to JAK2 inhibition and results in inhibition of transcriptional regulation of certain NF-κB proinflammatory targets. The combination of both the antiapoptotic and the anti-inflammatory effect may be beneficial in AKI. Additional studies are needed to further characterize the molecular and structural basis of the anti-inflammatory action and to develop novel therapeutic compounds.

## Supporting Information

Figure S1 **Apaf-1 expression in murine embryonic fibroblasts (MEF) derived from Apaf-1 knock out mice and wild-type controls.** Apaf-1 gene (**A**) and protein expression (**B**) levels in MEF-Apaf-1^+/+^ and MEF-Apaf-1^−/−^ evaluated by PCR and Western blot, respectively.
(TIFF)

Figure S2 **QM56 inhibits CsA and Tweak/TNFα/INFγ-induced JAK2 activation.** JAK2 is activated by 10 µg/ml CsA or 100 ng/ml Tweak, 30 ng/ml TNFα, 30 U/ml IFNγ in MCT cells and QM56 prevented this effect. Representative Western blot of three independent experiments.
(TIFF)

Figure S3 **QM56 inhibits LPS-induced NF-κB activation and NF-κB proinflammatory activity.** MCP1 and Rantes mRNA (qRT-PCR) synthesis (**A**) and JAK2, Stat3 and phospho(Ser536)-p65 pathway (**B**) were activated by 1 µg/ml LPS added to MCT cells at the specified times and down-regulated by QM56 pretreatment. In A, results are expressed as the Mean±SD of three independent experiments. *p<0.01 vs Control, **p<0.05 and ^#^p<0.02 vs LPS. In B, figures are representative of three independent experiments. Protein bands in the sequence were arranged from non-consecutive lanes on the same membrane.
(TIFF)

Figure S4 **QM56 treatment in folic acid-induced AKI reduces the nuclear localization of phospho(Ser536)-p65 in renal tubules.** In AKI group, tubules with an increased nuclear expression of p65 (asterisk) also present an increased nuclear expression of phospho-p65 (Ser536), but not in kidney tubules from the QM56 treated group. p65 and phospho-p65 (Ser536) are detected by immunohystochemical staining in the same renal tubules. Original magnification ×200.
(TIFF)

Materials and Methods S1 (PDF)
