# Supplementary figures and images for: A Polymeric Nanomedicine Diminishes Inflammatory Events in Renal Tubular Cells
Source: PLoS One. 2013 Jan 2;8(1):e51992. doi: 10.1371/journal.pone.0051992 (PMC3534689; doi:10.1371/journal.pone.0051992)

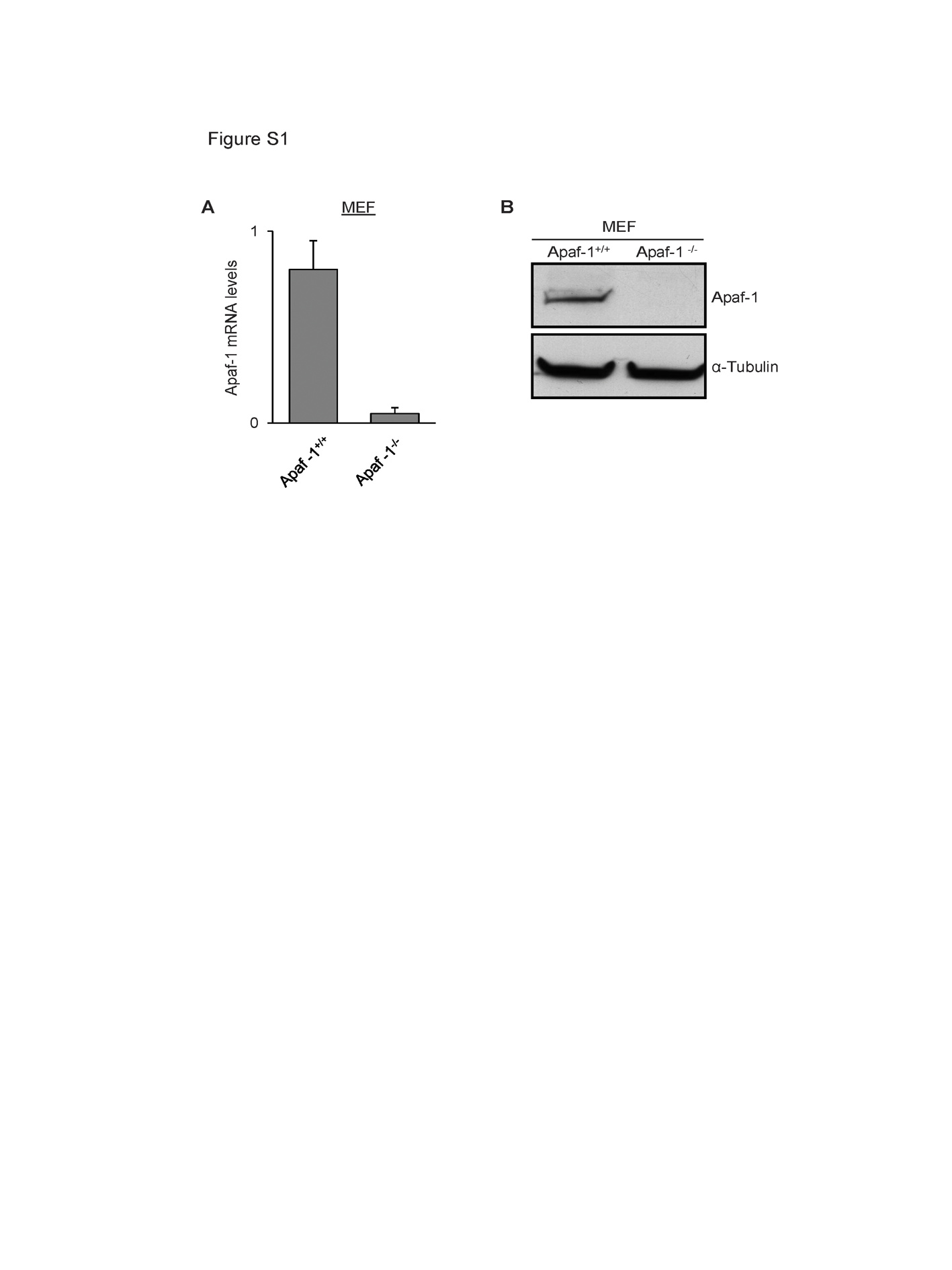

Supplement: Figure S1 — Apaf-1 expression in murine embryonic fibroblasts (MEF) derived from Apaf-1 knock out mice and wild-type controls. Apaf-1 gene (A) and protein expression (B) levels in MEF-Apaf-1+/+ and MEF-Apaf-1−/− evaluated by PCR and Western blot, respectively. (TIFF) [file pone.0051992.s001.tiff]

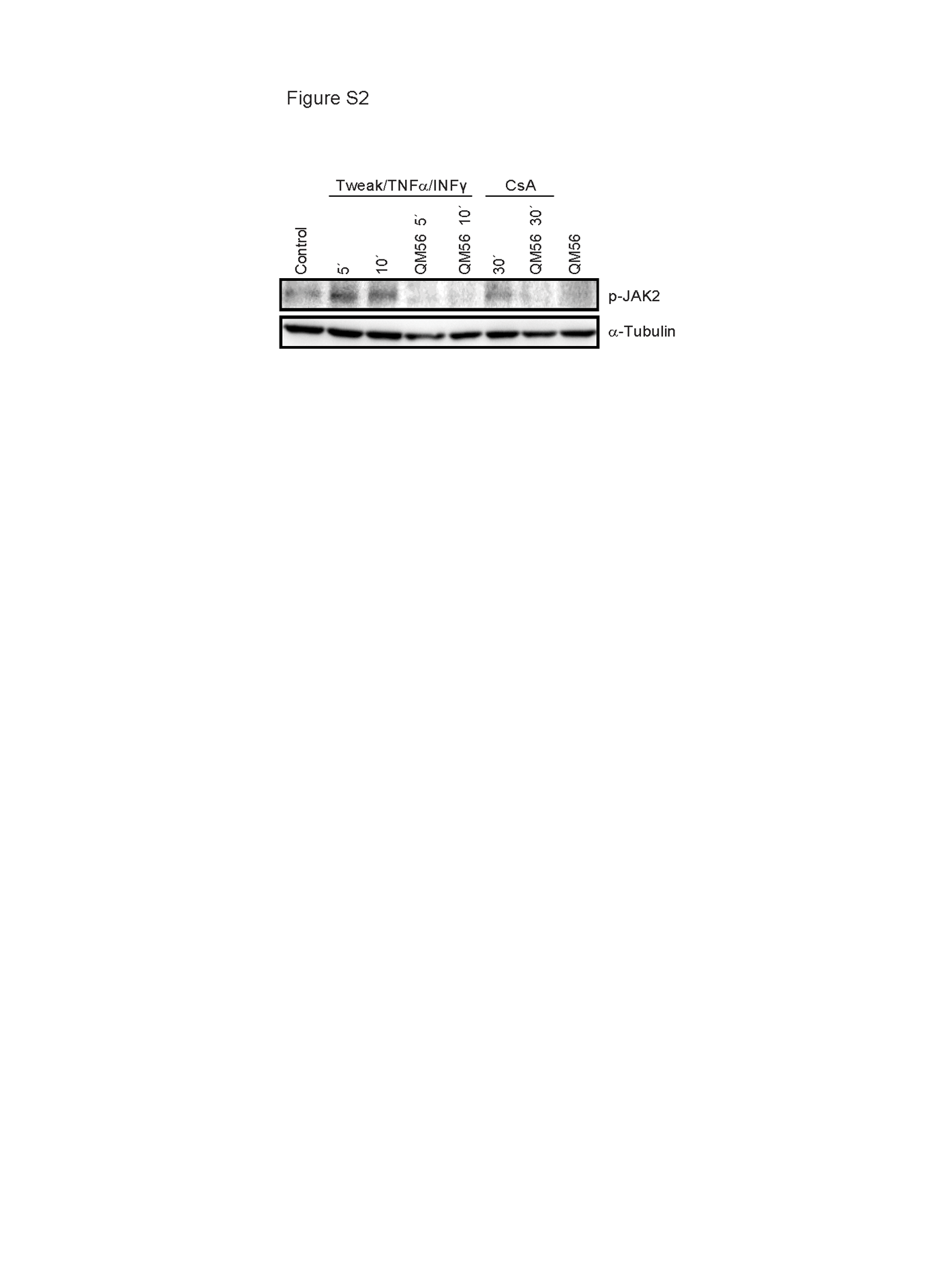

Supplement: Figure S2 — QM56 inhibits CsA and Tweak/TNFα/INFγ-induced JAK2 activation. JAK2 is activated by 10 µg/ml CsA or 100 ng/ml Tweak, 30 ng/ml TNFα, 30 U/ml IFNγ in MCT cells and QM56 prevented this effect. Representative Western blot of three independent experiments. (TIFF) [file pone.0051992.s002.tiff]

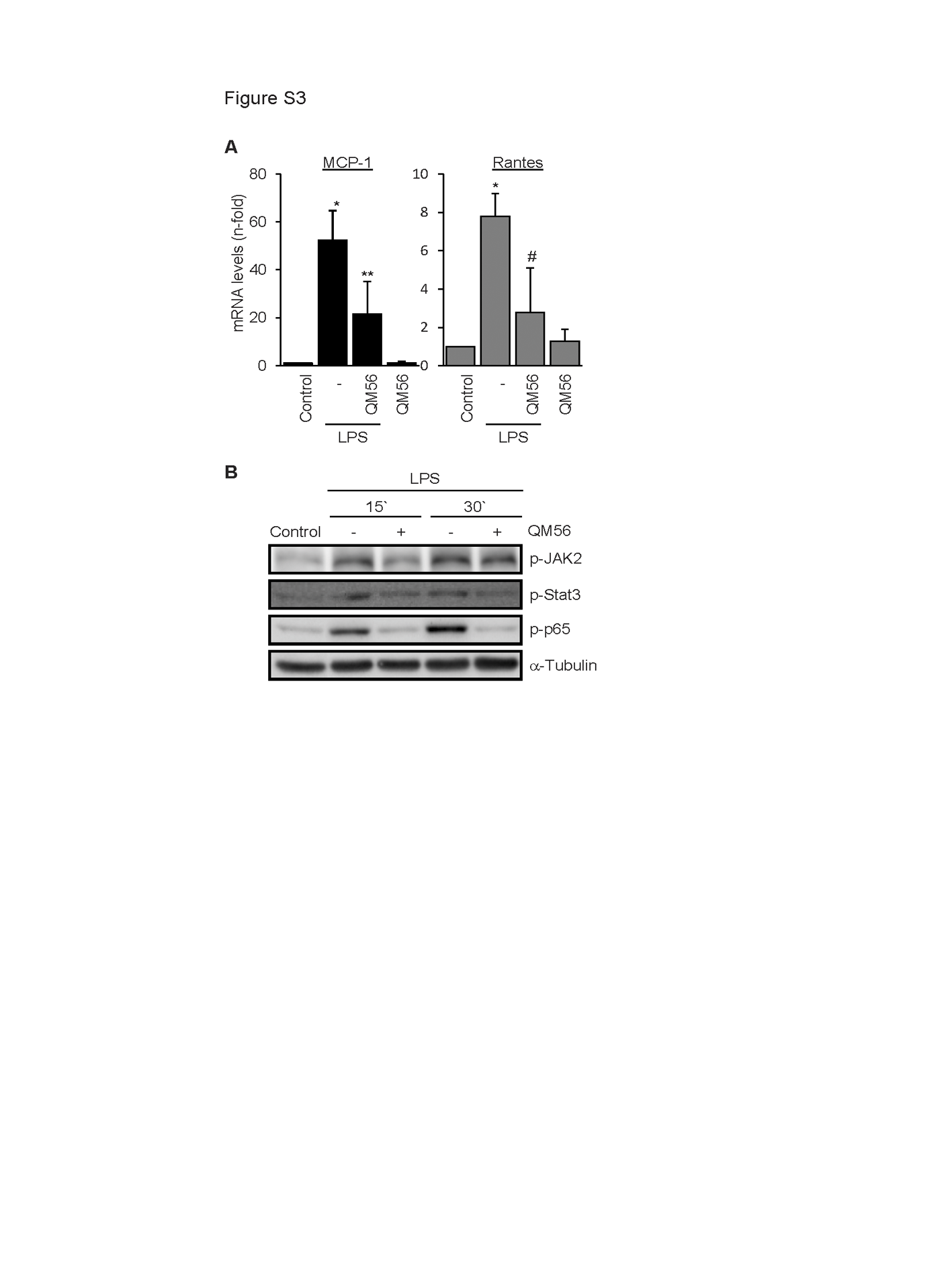

Supplement: Figure S3 — QM56 inhibits LPS-induced NF-κB activation and NF-κB proinflammatory activity. MCP1 and Rantes mRNA (qRT-PCR) synthesis (A) and JAK2, Stat3 and phospho(Ser536)-p65 pathway (B) were activated by 1 µg/ml LPS added to MCT cells at the specified times and down-regulated by QM56 pretreatment. In A, results are expressed as the Mean±SD of three independent experiments. *p<0.01 vs Control, **p<0.05 and #p<0.02 vs LPS. In B, figures are representative of three independent experiments. Protein bands in the sequence were arranged from non-consecutive lanes on the same membrane. (TIFF) [file pone.0051992.s003.tiff]

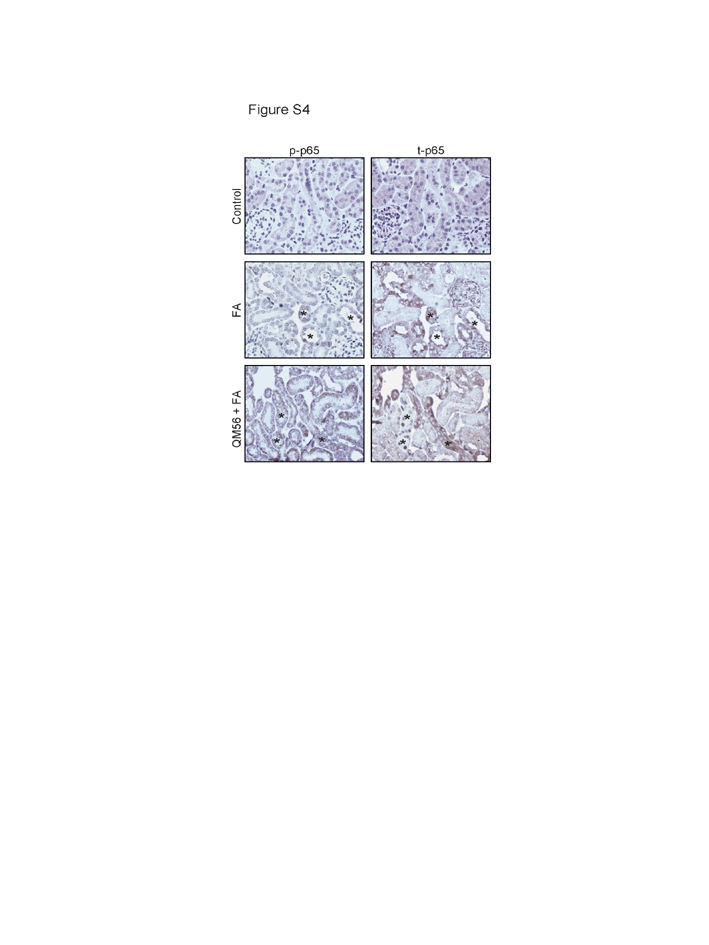

Supplement: Figure S4 — QM56 treatment in folic acid-induced AKI reduces the nuclear localization of phospho(Ser536)-p65 in renal tubules. In AKI group, tubules with an increased nuclear expression of p65 (asterisk) also present an increased nuclear expression of phospho-p65 (Ser536), but not in kidney tubules from the QM56 treated group. p65 and phospho-p65 (Ser536) are detected by immunohystochemical staining in the same renal tubules. Original magnification ×200. (TIFF) [file pone.0051992.s004.tiff]
