# Supplementary material for: A Polymeric Nanomedicine Diminishes Inflammatory Events in Renal Tubular Cells
Source: PLoS One. 2013 Jan 2;8(1):e51992. doi: 10.1371/journal.pone.0051992 (PMC3534689; doi:10.1371/journal.pone.0051992)
Supplement: Materials and Methods S1 — (PDF) [file pone.0051992.s005.pdf]

SUPPORTING MATERIALS AND METHODS

Cell cultures

MCT cells were cultured in RPMI 1640 (GIBCO, Grand Island, NY), 10% decomplemented fetal calf serum (FCS), 2 mmol/L, glutamine, 100 Um/L penicillin and 100 µg/ml streptomycin, in 5% CO_2 at 37°C. MEF-Apaf-1$^{-/-}$ and wild-type controls (MEF-Apaf-1$^{+/+}$) were grown in DMEM with the same additives as RPMI.

Synthesis of PGA-peptoid 1 conjugate QM56

Poly(L-glutamic acid) (PGA) sodium salt (0.35 g) with a molecular weight (Mw) range 17000-43000 Da was firstly transformed to PGA in its proton form. The pH of the aqueous PGA sodium salt solution was adjusted to 2.0 using 0.2 M HCl. The precipitate was collected, dialyzed against water, and lyophilized to yield 0.25 g. To a solution of PGA (75 mg, repeating unit FW 170, 0.44 mmol) in dry N,N-dimethylformamide (DMF) (8 mL) diisopropylcarbodiimide (DIC) (26 mL, 0.16 mmol) was added and after 5 min 4-hydroxybenzotriazole (HOBt) (20.4 mg, 0.16 mmol) as solid and left stirring for 15 min. Then NH_2-GlyGly-Peptoid 1 (44 mg, 0.052 mmol, molar ratio PGA/peptoid=8.5) previously synthesised by Fmoc-based solid phase synthesis, was added and the pH adjusted to 8 with diisopropylethylamine (DIEA). The reaction was allowed to proceed at room temperature for 36 h. Thin layer chromatography (TLC, silica) showed complete conversion of NH_2-GlyGly-Peptoid 1 (Rf=0.4) to polymer conjugate (Rf=0, CH_2Cl_2/MeOH=90:10). To stop the reaction, the mixture was poured into $CHCl_3$. The resulting precipitate was collected and dried in vacuum to yield the desired polymer-drug conjugate. The sodium salt of PGA-peptoid conjugate was obtained by dissolving the product in 1.0 M NaHCO3. This aqueous solution was dialysed against distilled water (MWCO 6,000 Da) to remove low molecular weight contaminants and salt excesses. Lyophilization of the dialysate yielded the product as a white powder (92% yield). The total peptoid content in this polymeric conjugates as determined by UV (λ= 282 nm) was in the range of 25-30 % (w/w) (10-12 mol% functionalisation).

Cell death and apoptosis

10,000 cells were seeded in 24-well plates (Costar, Cambridge, MA) in 10% FCS RPMI overnight. Thereafter, they were arrested in serum-free medium for 24h and stimuli were added to subconfluent cells. Adherent cells were pooled with spontaneously detached cells. After centrifugation, the cells were incubated with 100 µg/ml propidium iodide, 0.05% Igepal, 10 µg/ml RNAse A in PBS at RT for at least 1 h. The percentage of apoptotic cells with decreased DNA staining (hypodiploid population) was calculated. In addition, nuclei of formalin-fixed cells were stained with DAPI (Vector Laboratories, Inc., Burlingame, CA) to observe typical morphological nuclear changes by fluorescent microscopy. In the animal model, the total number of TUNEL positive nuclei was quantified in 20 randomly chosen fields (40x) per kidney using Image Pro Plus Software (Media cybernetics, Bethesda, MD)[20].

Electrophoretic mobility shift assay (EMSA)

Cell pellets were dissolved in 10 mmol/L HEPES, pH 7.8, 15 mmol/L, KCl, 2 mmol/L $MgCl_2$, 0.1 mmol/L EDTA, 1 mmol/L dithiotheitol (DTT), 1 mmol/L Phenylmethylsulfonyl fluoride (PMSF) (Buffer A). Nuclei and cytosolic fractions were separated by centrifugation at 1000×g 10 min. Nuclei (pellet) were washed twice in buffer A and resuspended in the same buffer with the aggregate of 0.39 mol/L KCl. Nuclei were extracted for 1h at 4ºC and centrifuged at 100,000×g 30 min. Supernatants were dialyzed against binding buffer containing 20 mmol/L HEPES-NaOH, pH 7.6, 20% (vol/vol) glycerol, 0.1 mmol/L NaCl, 5 mmol/L $MgCl_2$, 0.1 mmol/L EDTA, 1 mmol/L DTT, and 0.5 mmol/L PMSF (Buffer C) and then cleared by centrifugation and stored at –80ºC. The protein concentration was determined by the bicinchoninic acid method (Pierce Biotechnology, Rockford, IL).

Western blot

Protein content from cell extracts homogenized in lysis buffer (50 mmol/L Tris, 150 mmol/L NaCl, 2 mmol/L EDTA, 2 mmol/L EGTA, 0.2% Triton X-100, 0.3% NP-40, 0.1 mmol/L PMSF, 25 mmol/L NaF) was determined by the bicinchoninic acid method (Pierce Biotechnology). Nuclear and cytoplasmic fractionation were done with NE-PER® reagents (Thermo Scientific, Rockford, IL) according to manufacturer's instructions. Proteins were separated by 10% SDS-PAGE under reducing conditions and then blotted onto PVDF membranes. Membrane blockade was accomplished with 5% defatted milk in TBS-T (0.05 mol/L Tris, 0.15 mol/L NaCl, 0.05% Tween 20, pH 7.4). Thereafter, membranes were

overnight probed at 4ºC with specific primary antibodies made in the same blocking solution and then incubated with secondary HRP-conjugated antibodies for 1h at room temperature.
